# Shifting Patterns of Colorectal Cancer Burden in the United States (1999–2023): Implications for Precision Medicine Strategies and Drug Resistance in Early-Onset Colorectal Cancer

**DOI:** 10.3390/cancers18111768

**Published:** 2026-05-28

**Authors:** Chenyu Sun, Li Liu, Elizabeth H. Lees, Laura E. Billstein, Yuntao Zou, Abigail Fickel, Yan Yan, Yichen Wang, Tuan Vinh, Paul Travers, Vivek Kumbhari, Yuting Huang

**Affiliations:** 1 Division of Public Health, Infectious Diseases, and Occupational Medicine, Mayo Clinic, Rochester, MN 55905, USA; 2 Mayo Clinic School of Graduate Medical Education, Mayo Clinic College of Medicine and Science, Rochester, MN 55905, USA; 3 School of Public Health, University of Minnesota-Twin Cities, 420 Delaware St SE, Minneapolis, MN 55455, USA; 4 The Second People’s Hospital of Hefei, Guangde Road, Hefei 230011, China; 5 Hefei Hospital Affiliated to Anhui Medical University, Guangde Road, Hefei 230011, China; 6 Division of Hospital Medicine, Department of Medicine, University of California San Francisco, San Francisco, CA 94143, USA; 7 Mayo Clinic Alix School of Medicine, Mayo Clinic, Rochester, MN 55905, USA; 8 Gastroenterology & Hepatology, Mayo Clinic Florida, 4500 San Pablo Rd S, Jacksonville, FL 32224, USA; 9 Division of Medical Sciences, University of Oxford, Oxford OX3 9DU, UK

**Keywords:** colorectal cancer, early-onset cancer, precision medicine, drug resistance, health disparities, risk stratification

## Abstract

**Background**: Colorectal cancer (CRC) remains one of the common causes of cancer-related morbidity and mortality in the United States. Emerging increases in early-onset CRC and persistent disparities remain major public health concerns. **Objective**: To characterize long-term CRC incidence and mortality trends in the United States, with particular emphasis on identifying demographic groups experiencing rising or plateauing disease burden. **Methods**: We conducted a population-based ecological study using CRC incidence data from the United States Cancer Statistics database (1999–2022) and mortality data from CDC WONDER (1999–2023). Temporal trends were evaluated using Joinpoint regression to estimate annual percent change (APC) and average annual percent change (AAPC), stratified by age, sex, race/ethnicity, and urbanization level. **Results**: Overall CRC incidence and mortality declined during the study period (both AAPC approximately −2.08%). However, incidence increased significantly among adults younger than 55 years, particularly those aged 20–24 years. Mortality also increased among younger adults aged 25–44 years and remained elevated among individuals aged 45–54 years. Non-Hispanic Black individuals consistently experienced the highest incidence and mortality rates. Mortality declines plateaued in several older age groups after 2018–2020. **Conclusions**: Despite overall improvements in CRC burden, increasing incidence and mortality among younger adults and persistent disparities highlight the need for risk-stratified prevention, earlier detection strategies, and tailored therapeutic approaches.

## Simple Summary

Colorectal cancer rates have improved overall in the United States over the past two decades, largely due to improved screening and treatment. However, growing numbers of younger adults are being diagnosed with and dying from colorectal cancer, raising major public health concerns. In this study, we analyzed nationwide data from 1999 to 2023 to examine trends in colorectal cancer incidence and mortality across age, sex, race, ethnicity, and geographic groups. We found that while older adults experienced substantial improvements, colorectal cancer incidence and mortality increased among younger adults, particularly those under 55 years of age. Persistent disparities were also observed among Black individuals and people living in rural areas. These findings highlight the need for earlier detection strategies, equitable access to care, and more personalized approaches to prevention and treatment, including precision medicine strategies for younger and high-risk populations.

## 1. Introduction

Colorectal cancer (CRC) remains one of the top three most common causes of cancer-related morbidity and mortality in the United States [1]. In 2025 alone, it was estimated to account for 154,270 new cases and 52,900 deaths, underscoring its persistent public health impact [1]. Although overall CRC mortality trends have declined over the past few decades, largely due to advancements in screening (e.g., colonoscopy) and improvements in treatment (e.g., targeted therapy, immunotherapy) [2,3], these benefits have not been uniformly shared across the population [2,4]. Of particular concern is the steady rise in CRC incidence among adults younger than 50 years, a pattern that contrasts sharply with the declining burden in older age groups [1,5]. In addition, persistent disparities related to race, ethnicity, and socioeconomic status continue to contribute to unequal CRC burden [4,6]. In parallel with the increasing incidence of early-onset CRC, accumulating evidence suggests that younger patients frequently exhibit distinct molecular and genomic profiles, including alterations in mismatch repair pathways, KRAS signaling, BRAF status, CpG island methylation, and consensus molecular subtype (CMS) distribution [7,8]. These biologic differences may contribute not only to aggressive tumor behavior but also to heterogeneous responses to chemotherapy, targeted therapy, and immunotherapy [7,8]. Resistance to fluoropyrimidine-based chemotherapy, anti-EGFR therapy, and immune checkpoint inhibitors remains a major obstacle in CRC management [7,8], particularly in molecularly complex or underserved populations with limited resources. Consequently, understanding epidemiologic trends alongside evolving molecular characteristics has become increasingly important for guiding precision medicine strategies and overcoming treatment resistance.

Monitoring these evolving trends through reliable national databases is critical for informing prevention strategies, guiding the equitable deployment of therapeutic advances, and identifying populations who may benefit most from precision oncology approaches. The Centers for Disease Control and Prevention Wide-Ranging Online Data for Epidemiologic Research (CDC WONDER) platform offers a robust resource for such surveillance, providing population-based incidence and mortality data across multiple demographic and geographic dimensions [9,10]. Although a recent study by Eldesouki et al. used CDC WONDER and BRFSS data to examine CRC mortality, it focused solely on adults aged 45 years and older [11]. This study utilizes the most recent CDC WONDER data to systematically analyze temporal trends in CRC incidence (1999–2022) and mortality (1999–2023) among the adult population, including younger age groups, and to examine these trends stratified by key demographic and geographic variables such as age, sex, race/ethnicity, and urbanization level. By identifying populations with rising or stagnant mortality rates, this study aims to provide evidence to guide risk-stratified prevention and to inform the development of precision medicine approaches, including tailored screening, genomic characterization, targeted therapeutic strategies, and strategies to overcome drug resistance, for subgroups at highest risk of poor outcomes. In addition to conventional screening strategies, emerging biosensor-based technologies and minimally invasive diagnostic platforms are being actively developed to improve early CRC detection and population screening efficiency [12].

## 2. Materials and Methods

### 2.1. Study Design

This population-based ecological study examined temporal trends in colorectal cancer (CRC) incidence and mortality in the United States between 1999 and 2022/2023. Analyses were performed across multiple demographic and geographic subgroups, including age, sex, race/ethnicity, and urbanization level. Data were obtained from CDC WONDER, a publicly accessible de-identified database used for epidemiologic surveillance and population-level research [10]. Because the study used publicly available de-identified data, institutional review board approval and informed consent were not required.

### 2.2. Data Sources

Data were independently extracted by two investigators from the CDC WONDER platform. CRC incidence data were obtained from the United States Cancer Statistics (USCS) database (1999–2022), which integrates population-based cancer registry data from the National Program of Cancer Registries and the Surveillance, Epidemiology, and End Results (SEER) Program [13]. Mortality data were obtained from the CDC WONDER Underlying Cause of Death database (1999–2023), which is based on death certificate data compiled by the National Center for Health Statistics [14].

CRC cases and deaths were identified using the International Classification of Diseases, Tenth Revision (ICD-10) codes C18–C20, corresponding to malignant neoplasms of the colon, rectosigmoid junction, and rectum. The CDC WONDER platform provides annual counts, crude rates, age-adjusted rates, standard errors, and 95% confidence intervals (CIs), which can be stratified by demographic and geographic characteristics.

### 2.3. Variable Extraction and Subgroup Categorization

Incidence data from 1999–2022 and mortality data from 1999–2023 were extracted from the respective CDC WONDER databases. Incidence data were available only through 2022, whereas mortality data were available through 2023.

Demographic variables extracted for both incidence and mortality analyses included age, sex, and race/ethnicity. Age groups for incidence analyses were categorized into 5-year intervals beginning at 20 years of age (20–24, 25–29, etc.), whereas mortality analyses used 10-year age intervals beginning at 25 years of age (25–34, 35–44, etc.), consistent with data availability in the CDC WONDER database.

Race/ethnicity categories included Hispanic, non-Hispanic (NH) White, NH Black, and NH Other. The NH Other category included NH American Indian or Alaska Native and NH Asian or Pacific Islander populations for incidence analyses and earlier mortality datasets. Urbanization level was available only for mortality analyses from 1999–2020 and was categorized as metropolitan (large central metro, large fringe metro, medium metro, and small metro) or nonmetropolitan (micropolitan and noncore) according to CDC classification criteria [9,11].

### 2.4. Statistical Analysis

Temporal trends in CRC incidence and mortality were evaluated using Joinpoint regression analysis, a method commonly used in cancer epidemiology to identify significant changes in longitudinal trends [15,16]. Annual age-adjusted incidence rates (AAIRs) and age-adjusted mortality rates (AAMRs), standardized to the 2000 U.S. standard population, were extracted for each study year along with corresponding standard errors.

Joinpoint regression analyses were performed using the Joinpoint Regression Program version 5.1.0.0 (National Cancer Institute, Bethesda, MD, USA) [9,15,16]. Log-linear models were fitted to estimate annual percent changes (APCs) and average annual percent changes (AAPCs) with corresponding 95% confidence intervals (CIs). Joinpoints were identified using the permutation test model selection method. The parametric method was used for CI estimation, while all remaining settings were retained at default values.

For age-specific mortality analyses, age-adjusted mortality rates were unavailable in CDC WONDER; therefore, crude mortality rates were analyzed instead. In these analyses, the variable type was specified as “Crude Rate” within the Joinpoint software, Version 5.1.0.0 (National Cancer Institute, Bethesda, MD, USA), while all other analytical parameters remained unchanged.

Percent change between the initial and final study years was calculated as:

(Value in final year − Value in initial year)/Value in initial year × 100%

A two-sided *p* value < 0.05 was considered statistically significant. Data management and descriptive analyses were additionally performed using R software version 4.5.2 (R Foundation for Statistical Computing, Vienna, Austria).

## 3. Results

### 3.1. Overall Trends

Between 1999 and 2022/2023, colorectal cancer (CRC) incidence and mortality in the United States declined substantially overall. However, Joinpoint analysis demonstrated that the pace of improvement slowed after approximately 2012, particularly for mortality, which plateaued after 2020 (Table 1 and Table 2).

Overall CRC incidence decreased by 34.73% during the study period (AAPC = −2.08%, 95% CI: −2.32% to −1.78%), with the annual decline slowing from −2.71% during 1999–2012 to −1.24% during 2012–2022. Similarly, CRC mortality decreased by 38.96% overall (AAPC = −2.08%, 95% CI: −2.32% to −1.85%), but the decline attenuated over time and became non-significant after 2020 (Table 1 and Table 2).

### 3.2. Age-Specific Trends

**Figure 1:**
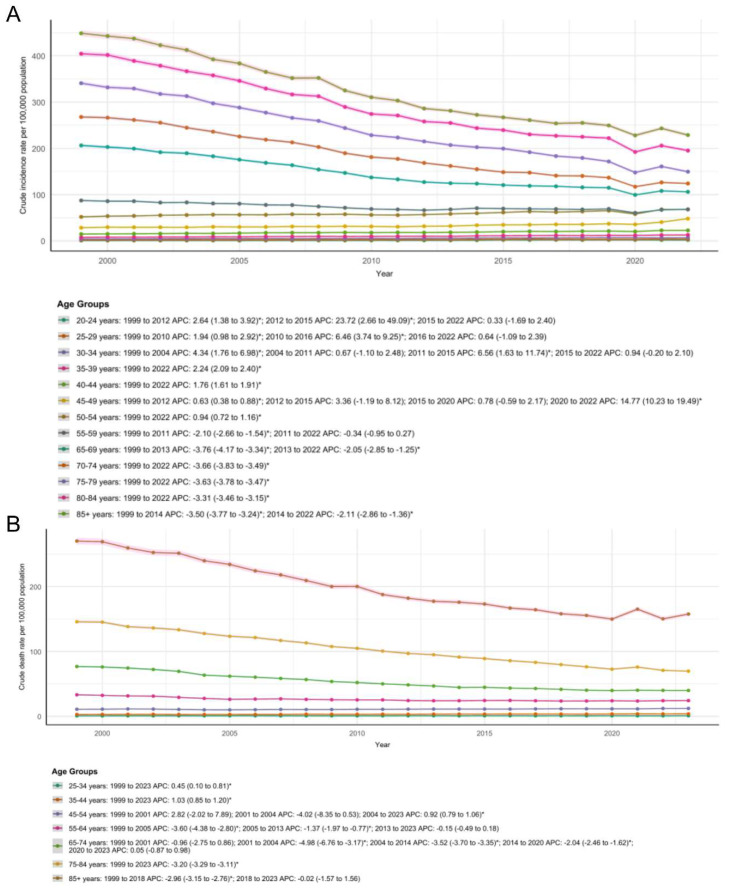
Temporal trends in colorectal cancer by age group in the United States. (**A**) Age-adjusted incidence rates (AAIRs). (**B**) Age-adjusted mortality rates (AAMRs). * indicates statistically significant.

Marked age-related differences were observed in CRC trends. Incidence and mortality increased among younger adults, whereas older age groups generally experienced declining rates, although these declines slowed in recent years (Table 1 and Table 2; Figure 1).

Significant increases in AAIR were observed for all age groups under 55. The largest relative increase was observed among individuals aged 20–24 years, whose incidence more than doubled during the study period. Sustained increases were also observed among adults aged 25–49 years, with particularly accelerated growth in the 45–49-year group after 2020. In contrast, incidence rates declined consistently among adults aged 55 years and older, with the steepest reductions observed in individuals aged 65–84 years (Table 1, Figure 1A).

Mortality trends largely mirrored incidence patterns. CRC mortality increased significantly among adults aged 25–44 years and continued to rise modestly among those aged 45–54 years. Although mortality declined among older adults, these improvements slowed substantially over time, with several older age groups showing recent stabilization or near-plateauing of mortality trends after 2018–2020 (Table 2, Figure 1B).

### 3.3. Sex Differences

**Figure 2:**
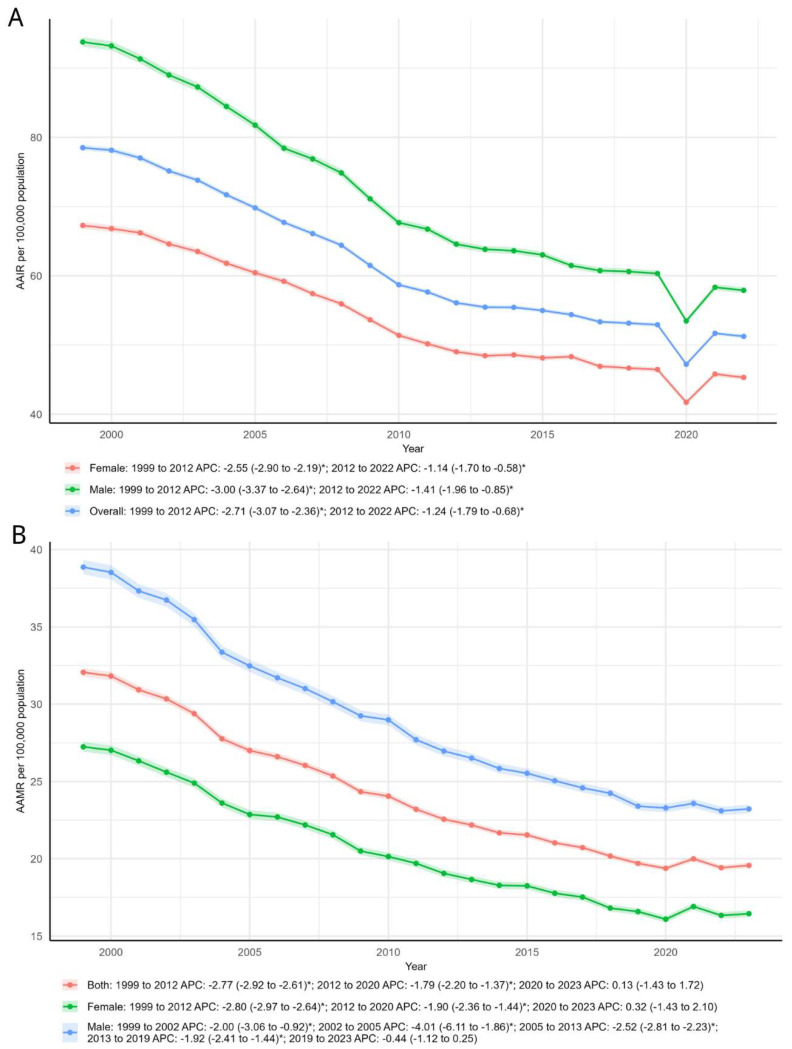
(**A**) Temporal trends of AAIR for CRC in the United States by sex; (**B**) Temporal trends of AAMR for CRC in the United States by sex. AAIR = age-adjusted incidence rate, AAMR = age-adjusted mortality rate, APC = annual percent change. * indicates statistically significant.

CRC incidence and mortality rates remained consistently higher in males than in females throughout the study period (Figure 2A,B). Although both sexes experienced substantial declines in incidence and mortality overall, the rate of improvement slowed after approximately 2012, and mortality trends plateaued in recent years (Table 1 and Table 2).

### 3.4. Racial and Ethnic Disparities

**Figure 3:**
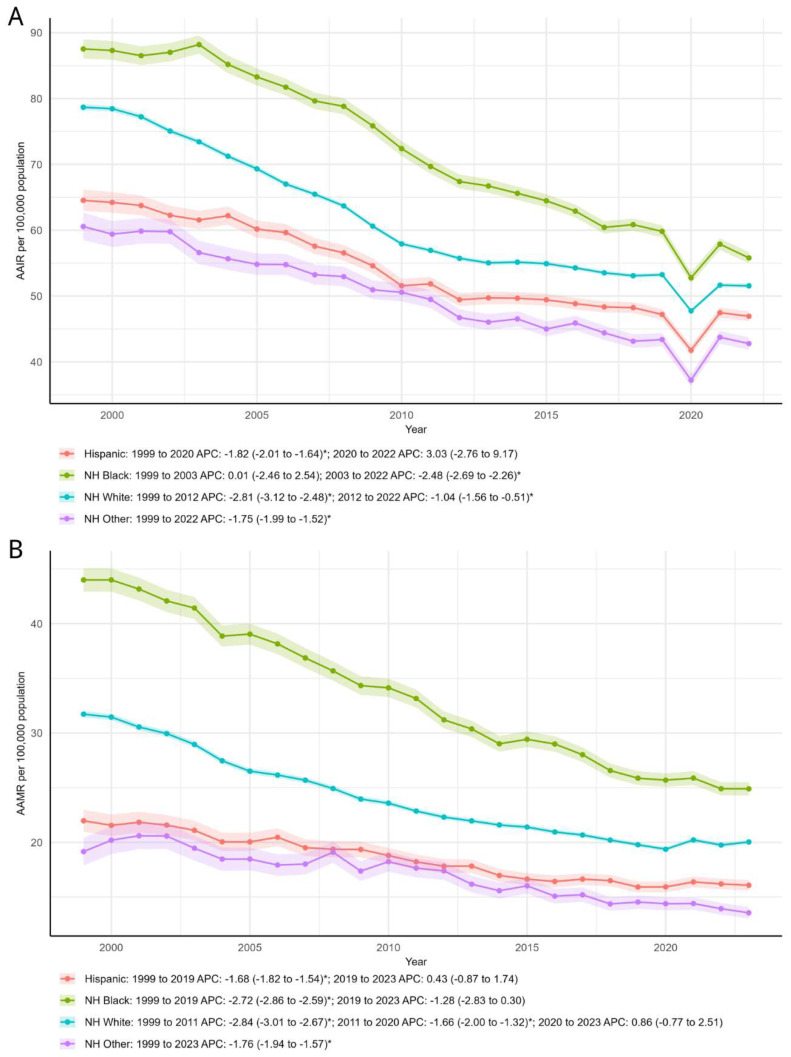
(**A**) Temporal trends of AAIR for CRC in the United States by race; (**B**) Temporal trends of AAMR for CRC in the United States by race. AAIR = age-adjusted incidence rate, AAMR = age-adjusted mortality rate, APC = annual percent change. * indicates statistically significant.

Non-Hispanic (NH) Black individuals consistently had the highest CRC incidence and mortality rates throughout the study period, despite experiencing substantial declines over time (Figure 3). Although racial disparities narrowed compared with 1999, NH Black populations continued to experience higher disease burden than NH White populations in 2022/2023. Hispanic and NH Other populations also demonstrated declining trends overall, although incidence temporarily decreased sharply around 2020 before rebounding thereafter. In recent years, mortality declines slowed or plateaued across most racial and ethnic groups (Table 1 and Table 2).

### 3.5. Urbanization Trends

CRC mortality declined in both metropolitan and nonmetropolitan areas between 1999 and 2020. However, nonmetropolitan areas consistently exhibited higher mortality rates and slower declines compared with metropolitan regions, indicating persistent urban-rural disparities in CRC outcomes (Table 2).

## 4. Discussion

This nationwide analysis demonstrated substantial declines in overall colorectal cancer (CRC) incidence and mortality in the United States from 1999 to 2022/2023. However, these favorable trends were accompanied by several concerning patterns, including increasing incidence and mortality among younger adults, slowing improvements in mortality after approximately 2012, and persistent racial and urban-rural disparities. These findings highlight the continuing public health burden of CRC and emphasize the importance of improving prevention, early detection, and equitable access to screening and treatment services.

### 4.1. Increasing Burden of Early-Onset CRC

The most notable finding of this study was the continued increase in CRC incidence among adults younger than 55 years. Several younger age groups demonstrated substantial increases over the study period, particularly those aged 20–24, 25–29, and 45–49 years. Mortality also increased among younger adults, especially in those aged 25–34 and 35–44 years, indicating that early-onset CRC is becoming both more common and more clinically significant.

These findings are consistent with previous reports from the United States and multiple other countries demonstrating a rising global burden of early-onset CRC, particularly in several high-income and transitioning countries. Similar increasing trends among younger adults have been reported in Europe, Canada, Australia, and parts of Asia, suggesting that this phenomenon is unlikely to be unique to the United States [17,18]. Potential contributing factors proposed in prior studies include increasing obesity prevalence, shifts toward high-fat and calorie-dense dietary patterns, increased alcohol consumption, sedentary lifestyles, metabolic disorders, and alterations in the gut microbiome, all of which have become more prevalent in younger generations [19,20,21,22,23]. These environmental and lifestyle-related factors may collectively influence colorectal carcinogenesis through complex metabolic and microbiome-related pathways. Although the mechanisms underlying these trends remain incompletely understood, the consistent rise across younger populations highlights the need for earlier prevention strategies, increased awareness of CRC symptoms in younger adults, and continued evaluation of screening recommendations. Future studies integrating molecular, genomic, and epidemiologic data may help clarify whether early-onset CRC represents a biologically distinct subgroup with unique risk profiles, therapeutic vulnerabilities, and implications for precision prevention and treatment strategies.

We also observed a sharp increase in incidence among adults aged 45–49 years during 2020–2022. This pattern may be the result of the updated CRC screening guideline in 2021 [24], and may also partially reflect delayed diagnosis and recovery of screening activity following disruptions during the COVID-19 pandemic, which affected CRC screening and diagnostic services in many regions [25,26].

### 4.2. Stalled Progress and Disparities: A Role for Molecularly Informed Equity

Despite overall improvements, substantial racial and geographic disparities persisted throughout the study period. Non-Hispanic Black individuals consistently experienced the highest incidence and mortality rates, although the absolute gaps compared with other racial groups narrowed over time. In addition, nonmetropolitan areas showed persistently higher mortality rates and slower declines compared with metropolitan areas.

These disparities likely reflect multiple interacting factors, including rising obesity rates, shifts toward high-fat and calorie-dense dietary patterns, increased alcohol consumption, suboptimal adherence to recommended screening among middle-aged adults, and lack of access to quality healthcare, socioeconomic conditions, comorbidity burden, and treatment availability [19,20,21,22,23,27,28,29,30,31,32,33,34,35,36]. Prior studies have also suggested that biologic and molecular heterogeneity may contribute to differences in CRC outcomes across populations [36,37,38], although such factors could not be evaluated in the present population-level analysis. Therefore, future studies integrating epidemiologic data with molecular and genomic profiling may help better clarify mechanisms underlying persistent disparities in CRC burden and treatment outcomes. The recent slowing or plateauing of mortality improvement in several populations after 2020 is also concerning and may partially reflect disruptions in healthcare utilization during the COVID-19 pandemic [25,26].

Overall, our findings underscore the importance of improving equitable access to CRC prevention, screening, and high-quality cancer care, particularly in historically underserved populations and rural communities. At the same time, more personalized and risk-adapted strategies incorporating both clinical and molecular risk factors may help improve outcomes in high-risk populations. Emerging artificial intelligence-based approaches may further enhance personalized CRC management by improving risk prediction, imaging interpretation, molecular classification, and treatment selection [39].

### 4.3. Limitations

This study has several important limitations. First, our analysis is based on death certificate data from CDC WONDER; therefore, the accuracy of our findings depends on the reliability and completeness of reporting. Misclassification of race, ethnicity, or cause of death may occur, and coding practices may vary over time. Second, the analysis relies on aggregated, population-level data and lacks individual-level clinical, behavioral, or molecular information, such as tumor stage, genetic markers, comorbidities, obesity, or screening history, which limits the ability to explore causal relationships or mechanisms underlying observed mortality trends. Third, suppression rules in the CDC WONDER database (e.g., <10 deaths) constrain trend estimation in smaller subpopulations [40], potentially attenuating apparent disparities. Fourth, our results primarily reflect U.S. populations during the study period and may not generalize to other countries or regions with different healthcare systems, risk-factor prevalence, or reporting practices. Finally, while mortality data for the most recent year may be provisional or incomplete, rigorous validation processes in CDC WONDER minimize this issue. Despite these limitations, the use of nationally representative population-level data provides valuable insight into long-term CRC trends and disparities in the United States.

## 5. Conclusions

Although the overall burden of colorectal cancer in the United States declined substantially between 1999 and 2022/2023, important challenges remain. Incidence and mortality increased among younger adults, improvements in mortality slowed after approximately 2012, and persistent racial and urban-rural disparities were observed. These findings highlight the need for strengthened prevention efforts, equitable access to screening and treatment, and continued evaluation of risk-adapted screening strategies. Future studies integrating molecular, genomic, and epidemiologic data may help better characterize biologic heterogeneity in early-onset CRC and support the development of more personalized prevention and treatment approaches. As similar increases in early-onset CRC have also been reported globally, the trends observed in the United States may reflect a broader international public health challenge requiring coordinated research and prevention efforts.

## Figures and Tables

**Table 1:** Trends in Colorectal Cancer Incidence in the United States, 1999–2022.

Variable	AAIR (per 100,000)			AAPC (95% CI)	Trend Periods & APC (95% CI)
	1999 (95% CI)	2022 (95% CI)	Percent Change (%)		
**Total**	78.50 (78.10–78.89)	51.24 (50.98–51.51)	−34.73	−2.08 (−2.37 to −1.78) *	1999–2012: −2.71 (−3.07 to −2.36) *; 2012–2022: −1.14 (−1. 79 to −0.68) *
**Sex**					
Male	93.77 (93.09–94.45)	57.89 (57.48–58.31)	−38.26	−2.31 (−2.61 to −2.02) *	1999–2012: −3.00 (−3.37 to −2.64) *; 2012–2022: −1.41 (−1.96 to −0.85) *
Female	67.27 (66.78–67.75)	45.31 (44.96–45.66)	−32.64	−1.94 (−2.23 to −1.64) *	1999–2012: −2.55 (−2.90 to −2.19) *; 2012–2022: −1.14 (−1.70 to −0.58) *
**Race**					
Hispanic	64.54 (62.94–66.16)	46.93 (46.19–47.67)	−27.29	−1.41 (−1.90 to −0.92) *	1999–2020: −1.82 (−2.01 to −1.64) *; 2020–2022: 3.03 (−2.76 to 9.17)
NH Black	87.53 (86.10–88.98)	55.79 (54.95–56.65)	−36.26	−2.05 (−2.48 to −1. 62) *	1999–2003: 0.01 (−2.46 to 2.54); 2003–2022: −2.48 (−2.69 to −2.26) *
NH White	78.68 (78.24–79.11)	51.55 (51.22–51.88)	−34.48	−2.04 (−2.31 to −1.77) *	1999–2012: −2.81 (−3.12 to −2.48) *; 2012–2022: −1.04 (−1.56 to −0.51) *
NH Other	60.55 (58.49–62.67)	42.78 (41.84–43.73)	−29.35	−1.75 (−1.99 to −1.52) *	1999–2022: −1.75 (−1.99 to −1.52) *
**Age Group (Years)**					
20–24	0.74 (0.62–0.87)	1.96 (1.78–2.15)	+165.86	4.45 (1.96 to 7.00) *	1999–2012: 2.64 (1.38 to 3.92) *; 2012–2015: 23.72 (2.66 to 49.09) *; 2015–2022: 0.33 (−1.69 to 2.40)
25–29	1.92 (1.73–2.12)	3.61 (3.37–2.87)	+88.21	2.76 (1.88 to 3.65) *	1999–2010: 1.94 (0.98 to 2.92) *; 2010–2016: 6.46 (3.74 to 9.25) *; 2016–2022: 0.64 (−1.09 to 2.39)
30–34	3.82 (3.56–4.10)	6.68 (6.35–7.02)	+74.96	2.55 (1.46 to 3.65) *	1999–2004: 4.34 (1.76 to 6.98) *; 2004–2011: 0.67 (−1.10 to 2.48); 2011–2015: 6.56 (1.63 to 11.74) *; 2015–2022: 0.94 (−0.20 to 2.10)
35–39	7.37 (7.02–7.73)	12.79 (12.32–13.27)	+73.46	2.24 (2.09 to 2.40) *	1999–2022: 2.24 (2.09 to 2.40) *
40–44	14.64 (14.13–15.15)	22.64 (22.01–23.28)	+54.68	1.76 (1.61 to 1.91) *	1999–2022: 1.76 (1.61 to 1.91) *
45–49	28.33 (27.59–29.09)	48.01 (47.05–48.99)	+69.48	2.18 (1.48 to 2.88) *	1999–2012: 0.63 (0.38 to 0.88) *; 2012–2015: 3.36 (−1.19 to 8.12); 2015–2020: 0.78 (−0.59 to 2.17); 2020–2022: 14.77 (10.23 to 19.49) *
50–54	51.79 (50.71–52.89)	67.80 (66.69–68.93)	+30.91	0.94 (0.72 to 1.16) *	1999–2022: 0.94 (0.72 to 1.16) *
55–59	87.20 (85.60–88.83)	68.17 (67.07–69.30)	−21.82	−1.26 (−1.65 to −0.88) *	1999–2011: −2.10 (−2.66 to −1.54) *; 2011–2022: −0.34 (−0.95 to 0.27)
60–64	136.16 (133.94–138.41)	81.79 (80.58–83.02)	−39.92	−2.35 (−2.66 to −2.03) *	1999–2012: −3.43 (−3.88 to −2.97) *; 2012–2022: −1.15 (−1.66 to −0.65) *
65–69	206.18 (203.29–209.10)	105.93 (104.46–107.41)	−48.62	−3.09 (−3.47 to −2.72) *	1999–2013: −3.76 (−4.17 to −3.34) *; 2013–2022: −2.05 (−2.85 to −1.25) *
70–74	267.71 (264.30–271.16)	123.91 (122.15–125.69)	−53.71	−**3.66 (**−**3.83 to** −3.49) *	1999–2022: −3.66 (−3.83 to −3.49) *
75–79	340.74 (336.50 to 345.01)	149.48 (147.19–151.80)	−56.13	−3.63 (−3.78 to −3.47) *	1999–2022: −3.63 (−3.78 to −3.47) *
80–84	404.44 (398.78–410.16)	195.08 (191.72–198.49)	−51.76	−**3.31 (**−**3.46 to** −3.15) *	1999–2022: −3.31 (−3.46 to −3.15) *
85+	448.59 (442.13–455.13)	228.66 (224.87–232.50)	−49.03	−**3.02 (**−**3.31 to** −2.73) *	1999–2014: −3.50 (−3.77 to −3.24) *; 2014–2022: −2.11 (−2.86 to −1.36) *

Abbreviations: AAIR = age-adjusted incidence rate; AAPC = average annual percent change; APC = annual percent change; CI = confidence interval; NH = non-Hispanic. Notes: * *p* < 0.05 (statistically significant). Data source: CDC WONDER. United States and Puerto Rico Cancer Statistics. Bold values indicate statistically significant results.

**Table 2:** Trends in Colorectal Cancer Mortality in the United States, 1999–2023.

Variable	AAMR (per 100,000)			AAPC (95% CI)	Trend Periods (APC, 95% CI)
	1999 (95% CI)	2023 (95% CI)	Percent Change (%)		
**Total**	32.06 (31.80–32.33)	19.57 (19.40–19.74)	−38.96	−2.08 (−2.32 to −1.85) *	1999–2012: −2.77 (−2.92 to −2.61) *; 2012–2020: −1.79 (−2.20 to −1.37) *; 2020–2023: 0.13 (−1.43 to 1.72)
**Sex**					
Male	38.87 (38.41–39.33)	23.23 (22.95–23.50)	−40.25	−2.15 (−2.47 to −1.83) *	1999–2002: −2.00 (−3.06 to −0.92) *; 2002–2005: −4.01 (−6.11 to −1.86) *; 2005–2013: −2.52 (−2.81 to −2.23) *; 2013–2019: −1.92 (−2.41 to −1.44) *; 2019–2023: −0.44 (−1.12 to 0.25)
Female	27.25 (26.93–27.56)	16.45 (16.24–16.66)	−39.63	−2.12 (−2.38 to −1.86) *	1999–2012: −2.80 (−2.97 to −2.64) *; 2012–2020: −1.90 (−2.36 to −1.44) *; 2020–2023: 0.32 (−1.43 to 2.10)
**Race**					
Hispanic	21.98 (20.97–22.99)	16.08 (15.62–16.54)	−26.84	−1.33 (−1.56 to −1.10) *	1999–2019: −1.68 (−1.82 to −1.54) *; 2019–2023: 0.43 (−0.87 to 1.74)
NH Black	44.00 (42.93–45.07)	24.90 (24.30–25.50)	−43.41	−2.49 (−2.75 to −2.22) *	1999–2019: −2.72 (−2.86 to −2.59) *; 2019–2023: −1.28 (−2.83 to 0.30)
NH White	31.72 (31.44–32.01)	20.04 (19.83–20.24)	−36.84	−1.94 (−2.18 to −1.71) *	1999–2011: −2.84 (−3.01 to −2.67) *; 2011–2020: −1.66 (−2.00 to −1.32) *; 2020–2023: 0.86 (−0.77 to 2.51)
NH Other	19.15 (17.88–20.42)	13.56 (13.04–14.08)	−29.20	−1.76 (−1.94 to −1.57) *	1999–2023: −1.76 (−1.94 to −1.57) *
**Age Group (Years)**					
25–34	0.69 (0.61–0.77)	0.81 (0.72–0.89)	+16.89	0.45 (0.10 to 0.81) *	1999–2023: 0.45 (0.10 to 0.81) *
35–44	2.87 (2.72–3.03)	3.71 (3.53–3.88)	+28.99	1.03 (0.85 to 1.20) *	1999–2023: 1.03 (0.85 to 1.20) *
45–54	10.88 (10.55–11.22)	12.14 (11.80–12.48)	+11.54	0.45 (−0.22 to 1.11)	1999–2001: 2.82 (−2.02 to 7.89); 2001–2004: −4.02 (−8.35 to 0.53); 2004–2023: 0.92 (0.79 to 1.06) *
55–64	33.17 (32.43–33.90)	24.26 (23.79–24.73)	−26.86	−1.43 (−1.72 to −1.13) *	1999–2005: −3.60 (−4.38 to −2.80) *; 2005–2013: −1.37 (−1.97 to −0.77) *; 2013–2023: −0.15 (−0.49 to 0.18)
65–74	76.81 (75.54–78.07)	39.84 (39.18–40.51)	−48.13	−2.69 (−2.97 to −2.40) *	1999–2001: −0.96 (−2.75 to 0.86); 2001–2004: −4.98 (−6.76 to −3.17) *; 2004–2014: −3.52 (−3.70 to −3.35) *; 2014–2020: −2.04 (−2.46 to −1.62) *; 2020–2023: 0.05 (−0.87 to 0.98)
75–84	145.84 (143.70–147.98)	69.63 (68.42–70.83)	−52.26	−3.20 (−3.29 to −3.11) *	1999–2023: −3.20 (−3.29 to −3.11) *
85+	270.27 (265.27–275.27)	157.66 (154.53–160.79)	−41.67	−2.35 (−2.68 to −2.02) *	1999–2018: −2.96 (−3.15 to −2.76) *; 2018–2023: −0.02 (−1.57 to 1.56)
Urban-rural **					
Metropolitan	18.30 (17.00–19.60)	13.92 (13.31–14.53)	−23.93	−1.79 (95% CI: −2.05 to −1.53) *	1999 to 2020 APC: −1.79 (−2.05 to −1.53) *
Nonmetropolitan	27.89 (22.77–33.01)	20.57 (17.84–23.30)	−26.25	−1.17 (95% CI: −1.64 to −0.69) *	1999 to 2020 APC: −1.17 (−1.64 to −0.69) *

Abbreviations: AAMR = age-adjusted mortality rate; AAPC = average annual percent change; APC = annual percent change; CI = confidence interval; NH = non-Hispanic. Notes: * *p* < 0.05 (statistically significant). Data source: CDC WONDER Underlying Cause of Death Database. ** Urban-rural analysis from 1999–2020, with the final year of 2020 instead of 2023. Bold values indicate statistically significant results.

## Data Availability

Data were obtained from the publicly available CDC WONDER.
